# An unusual intragenic promoter of *PIWIL2* contributes to aberrant activation of oncogenic *PL2L60*

**DOI:** 10.18632/oncotarget.17553

**Published:** 2017-05-02

**Authors:** Shan-Shan Liu, Ning Liu, Meng-Yao Liu, Lei Sun, Wu-Yan Xia, Hong-Min Lu, Yu-Jie Fu, Guo-Liang Yang, Juan-Jie Bo, Xiao-Xing Liu, Haizhong Feng, Hailong Wu, Lin-Feng Li, Jian-Xin Gao

**Affiliations:** ^1^ State Key Laboratory of Oncogenes and Related Genes, Renji-Med X Clinical Stem Cell Research Center, Ren Ji Hospital, School of Medicine, Shanghai Jiao Tong University, Shanghai, China; ^2^ Department of Urology, Ren Ji Hospital, School of Medicine, Shanghai Jiao Tong University, Shanghai, China; ^3^ Department of Radiotherapy, Ren Ji Hospital, School of Medicine, Shanghai Jiao Tong University, Shanghai, China

**Keywords:** PIWIL2, PL2L60, intragenic promoter, alienated gene activation, STAT3

## Abstract

PIWIL2-like (PL2L) protein 60 (PL2L60), a product of aberrantly activated *PIWIL2* gene, is widely expressed in various types of tumors and may promote tumorigenesis. However, the mechanisms underlying the activation of expression of *PL2L60* remain unknown. In this study, an intragenic promoter responsible for the activation of *PL2L60* within the human *PIWIL2* gene has been identified, cloned and characterized. The promoter of *PL2L60* is located in the intron 10 of the host gene *PIWIL2*. Bioinformatic and mutagenic analysis reveals that this intragenic promoter within the sequence of 50 nucleotides contains two closely arranged cis-acting elements specific for the hepatic leukemia factor (HLF) in the positive strand and signal transducer and activator of transcription 3 (STAT3) in the negative strand. Chromatin immunoprecipitation analysis demonstrates that both the HLF and polymerase II (Pol II), a hallmark of active promoters, directly bind to the sequence, although STAT3 does not. Knockdown of HLF and STAT3 alone or both by RNA interference significantly reduced both promoter activity and the PL2L60 protein expression, although there is no additive effect. The expression of PL2L60 proteins was enhanced when host gene *Piwil2* was genetically disrupted in a murine cell model. Taken together, we have identified a PL2L60-specific intragenic promoter in the host gene of *PIWIL2*, which is interdependently activated by HLF and STAT3 through steric interaction. This activation is dependent on cellular *milieu* rather than the integrity of host gene *PIWIL*2, highlighting a novel, important mechanism for a cancer-causing gene to be activated during tumorigenesis.

## INTRODUCTION

The phenomenon of aberrant activation has been observed in *PIWIL2* (piwi-like RNA-mediated gene silencing 2) gene in the tumorigenic status [1, 2]. The *PIWIL2* (alias *Mili* in mice, *HILI* in humans) is a member of P-element-induced wimpy testis/Argonaute (PIWI/AGO) gene family [3–6], mediating gematogenesis and tumorigenesis [2, 5, 7–12]. The *PIWIL2* is usually expressed in testis [4–6], but can be activated in somatic cells upon DNA damages to promote DNA repair through remodeling chromatin [13]. It plays crucial roles in self-renewal and maintenance of germline stem cells [3, 14], transposon repression via methylation [15, 16], chromatin remodeling [17, 18], biogenesis of piwi-interacting RNA [19] and translational regulation [14] in various organisms during development. However, the expression of PIWIL2 has been observed in various types of primary cancers and tumor cell lines [1, 11], including breast cancer [20], cervical cancer [21], gastric cancer [22],acute myeloid leukemia [23], colorectal cancer [24], colon cancer [24, 25], ovarian cancer [26] and testicular germ cell tumors [11, 27], etc.

We and others have found that PIWIL2 has multiple variants including PL2L80, PL2L80A, PL2L60, PL2L60A, PL2L50 and PL2L40. Some of the variants appear to be transcribed by intragenic promoters rather than a canonical promoter [1, 2, 27]. While full length PIWIL2 can mediate DNA repair acting as a barrier gene to the initiation of tumorigenesis and promote apoptotic cell death in tumor tissues [1, 13, 28], its variants such as PL2L60 [1] and PL2L60A [27] can promote tumorigenesis. Interestingly, these variants appear not to be derived from canonical splicing of full length mRNAs of *PIWIL2* gene rather being directly produced from short form of mRNAs transcribed by putative intragenic promoters in the host gene of *PIWIL2* [1, 27]. Since the variants such as PL2L60 has opposite functions to full length PIWIL2 in tumor development, e.g., tumor promoting vs. tumor suppression [1, 13, 28], we have referred to the intragenic promoter-mediated activation as aberrant or alienated activation of host gene [1, 2]. Among the variants mentioned above, PL2L60 is predominantly expressed in precancerous stem cells (pCSCs) as well as in various types of human and murine tumor cell lines with a level much higher than full length of PIWIL2 [1, 8]. PL2L60 can promote tumor cell survival and proliferation *in vitro* through up-regulation of *STAT3* and *BCL2* genes. It can also coordinate with NF-κB to promote tumorigenesis, probably representing a common pathway for the development of tumors in various types of tissues [1, 17, 28]. Importantly, peptides derived from PL2L60 can serve as strong immunogens targeting various types of cancers [1, 29]. Interestingly, while Piwil2, PL2L80 and PL2L60 were detected in the testicular cells of mice, knockout of *Piwil2* by homologous recombination did not abrogate the expressions of PL2L60 and PL2L80 in the testis [1], implicating that *PL2L60* and *PL2L80* might be transcribed by alternative promoters in the host gene *Piwil2*.

Alternative promoters may exist in intergenic or intragenic regions [30, 31]. Intragenic promoters have been described for transcription of tRNA and other non-coding (nc) RNAs [32, 33] as well as protein-coding RNAs [31, 34–38]. Recently, it has been reported that intragenic enhancers may act as alternative promoters transcribing multiple exonic poly(A)^+^ RNA (meRNA) from host gene, although the function of meRNA remains unclear [39].

In this study, we have cloned and characterized a human intragenic promoter that resides in the intron 10 of the host gene *PIWIL2*, which is responsible for the transcription of mRNA encoding PL2L60 proteins in testicular cells and tumor cells [1, 2]. To distinguish from spliced variants of mRNAs transcribed by canonical promoter and from the recently reported meRNAs transcribed by intragenic enhancers [39], we referred to this class of mRNAs transcribed by intragenic promoters as aberrant mRNA (amRNA). The protein product of amRNA may be opposite to that of host gene in functions, especially in tumor cells. The *PL2L60*-specific promoter may be bound by multiple transcription factors such as the hepatic leukemia factor (HLF) and signal transducer and activator of transcription 3 (STAT3) that might be not tightly bound to cis-acting elements. The cellular *milieu* rather than the integrity of host genes may determine the activation status of intragenic promoter. Our results suggest that activation of intragenic promoter might be a driving force for aberrant activation of host genes, representing an important mechanism underlying the development and tumorigenesis.

## RESULTS

### Cloning *PL2L60*-specific intragenic promoter in human *PIWIL2* gene

**Figure 1:**
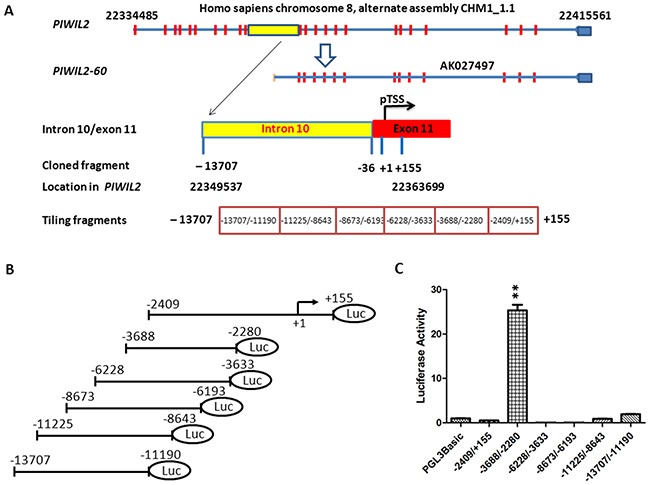
Identification of *PL2L60*-specific intragenic promoter in the host gene of *PIWIL2* **(A)** *Schematic diagram of the predicted region of intragenic promoter specific for PL2L60 in human PIWIL2 genes*: Full length *PIWIL2* gene is located in chromosome 8 range from 22334485 to 22415561 (alternate assembly CHM1_1.1). Based on the PL2L60 mRNA (AK027497) [1], putative transcriptional start site (pTSS) (+1) was determined and a range of nucleotides (nt) from 22349537 to 22363699 was cloned (−13707/+155) and divided into six tiling fragments: -2409/+155, -3688/−2280, -6228/−3633, -8673/−6193, -11225/−8643 and -13707/−11190. **(B)** *Structure of vectors harboring relevant fragments*: Each tiling fragment was cloned into a pGL3-basic luciferase report vector. **(C)** *Compare luciferase activity of the vectors containing tiling fragments*: Each vector was transfected into HEK-293T cells and luciferase activity was measured 48 h later. Only does the vector harboring the fragment -3688/−2280 display strong luciferase activity. The data shown are the luciferase activity from three independent experiments (mean ± SE). Yellow square: part of intron 10; **, p < 0.01 and ***, p < 0.001, as compared to control and determined by Student T-test.

We have found that while PIWIL2 is mainly detected in apoptotic cells of primary cancers, PL2L60, a variant of PIWIL2, is widely expressed in various types of proliferating cancer cells, promoting tumor growth [1]. Using the Gene2Promoter software from Genomatix Software Inc. (Ann Arbor, MI), we have predicted that the promoter of *PL2L60* gene encoding PL2L60 proteins is located in the region of the intron between exon 10 and 11 of *PIWIL2* gene. This promoter is supposed to initiate transcription of PL2L60 mRNA (AK027497), which can be translated into an about 60 kDa protein (PL2L60) [1]. To further elucidate the mechanism underlying PL2L60 expression, we attempted to clone the putative intragenic promoter specific for *PL2L60* within the host gene of *PIWIL2*. Based on the bioinformatic analysis, we defined a potential promoter region of *PL2L60* within the region consisting of the intron 10 and partial sequence of exon 11 in *PIWIL2* [1]. The predicted PL2L60 amRNA (AK027497) is the same as the sequence starting from 36^th^ nucleotide (nt) of exon 11 to exon 23 of *PIWIL2* gene (Figure 1A). To clone *PL2L60*-specific promoter, we defined the first nt of the predicted PL2L60 amRNA as the putative transcriptional start site (pTSS; +1) located at the 36^th^ base of exon 11 of *PIWIL2* (Genomic location: Homo sapiens chromosome 8, alternate assembly CHM1_1.1: 22334485-22415561; in the *PIWIL2* gene: 22361165-22361214; Figure 1). The nucleotide sequence from +155 to -13,707, equivalent to the location from 22349537 to 22363699 of chromosome 8, was used to screen *PL2L60* promoter (Figure 1A). The fragment was divided into six tiling fragments, amplified by PCR (Supplementary Table 1). A series of PL2L60-luciferase (p60-Luc) promoter reporting vectors (V-2409/+155, V-3688/−2280, V-6228/−3633, V-8673/−6193, V-11225/−8643, and V-13707/−11190) were constructed, using pGL3-basic vectors inserted with tiling fragments (Figure 1A & 1B). Each constructed vector was transfected into human HEK-293T cells and the cells were harvested and lysed 48 hrs later. The luciferase activity of each construct was determined using the Dual-Luciferase Reporter Assay kit (Promega, USA). The pGL3-basic vectors without an exogenous insert served as a negative control (Figure 1C). Among the constructs, V-3688/−2280 displayed a significantly higher level of luciferase activity as compared to other vectors (Figure 1C). The luciferase activity of V-2409/+155, V-6228/−3633, V-8673/−6193, V-11225/−8643 and V-13707/−11190 was almost the same as or even lower than the pGL3-basic negative control vector (Figure 1C). The results indicated that the region between -3688 and -2280 in the intron 10 of *PIWIL2* gene might contain promoter activity specific for *PL2L60*.

### Distinguishing *PL2L60*-specific intragenic promoter from intragenic enhancer

**Figure 2:**
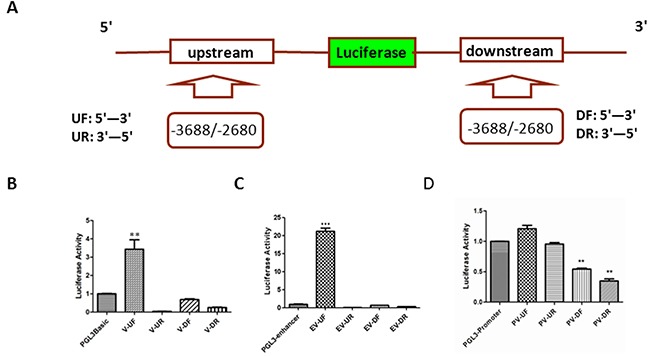
Distinguishing the intragenic promoter from enhancer **(A)** *Schematic diagram of construct for enhancer activity assay*: A series of vectors were constructed with the fragment -3688/−2680, which was cloned into pGL3-basic or pGL3-enhancer report vector up (U) or down (D) stream of luciferase gene in forward (F: 5′-3′) or reverse (R: 3′-5′) direction. UF: the fragment -3688/−2680 from 5′ to 3′ was cloned into upstream of the luciferase gene; UR: the fragment -3688/−2680 from 3′ to 5′ was cloned into upstream of the luciferase gene; DF: the fragment -3688/−2680 from 5′ to 3′ was cloned into downstream of the luciferase gene; and DR: the fragment -3688/−2680 from 3′ to 5′ was cloned into downstream of the luciferase gene. **(B)** *Luciferase activity of the fragment -3688/−2680 in the pGL3-basic report vectors (V)*: HEK-293T cells were transiently co-transfected with 1 μg V-UF, V-UR, V-DF or V-DR luciferase report vectors and 20 ng expression plasmids for Renilla luciferase (pRL-null). Transfection efficiency was normalized and the data shown are the luciferase activity from three experiments (mean ± SD). **, p < 0.01. **(C)** *Luciferase activity of the fragment -3688/−2680 in the pGL3-enhancer report vectors (EV)*: HEK-293T cells were transiently co-transfected with 1 μg EV-UF, EV-UR, EV-DF or EV-DR luciferase report vectors and 20 ng expression plasmids for Renilla luciferase (pRL-null). Transfection efficiency and luciferase activity was determined as described in **(B)** and the data shown are the luciferase activity from three experiments (Mean ± SD). ***, p < 0.001. **(D)** *Luciferase activity of the fragment -3688/−2680 in the pGL3-promoter report vectors (PV)*: HEK-293T cells were transiently co-transfected with 1 μg PV-UF, PV-UR, PV-DF or PV-DR luciferase report vectors and 20 ng expression plasmids for Renilla luciferase (pRL-null). Transfection efficiency and luciferase activity was determined as described in **(B)** and the data shown are the luciferase activity from three experiments (Mean ± SD). **, p < 0.01.

The promoter of a gene is usually located about 50 - 100 nt upstream of transcriptional start sites (TSS). Since the vector V-3688/−2280 with high luciferase activity was far away (1,409 nt) from the putative TSS (pTSS) of PL2L60, it might be associated with the activity of an intragenic enhancer, which usually regulates neighboring or distant genes but might act as a promoter to transcribe meRNAs of host genes [39]. Thus, we investigated whether the luciferase activity of V-3688/−2280 represented the activity of an intragenic enhancer. Since enhancer has bidirectional or reverse transcription activity, we constructed four vectors with the fragments-3688/−2680, which were amplified by PCR and inserted at the up-stream or down-stream of luciferase gene in pGL3-basic reporting vector in a direction of 5′ to 3′ and 3′ to 5′, respectively (Figure 2A; Supplementary Table 1). Then, these plasmid vectors (V-UF, V-UR, V-DF, and V-DR) were transfected into HEK-293T cells, respectively. After 48 hrs of transfection, the cells were lysed and the luciferase activity was determined as described above. As shown in Figure 2B, only the vector V-UF displayed significant luciferase activity when compared to that of pGL3-basic control vectors. The results indicated that the luciferase activity of fragment -3688/−2680 was unidirectional from 5′ – 3′ in forward direction but not in reverse direction up-stream of luciferase gene, suggesting that the activity of the fragment represented an intragenic promoter rather than an intragenic enhancer acting as an alternative promoter [39].

To further validate the results above, the fragment -3688/−2680 in forward or reverse direction was cloned into the upstream (EV-UF and EV-UR) or downstream (EV-DF and EV-DR) of luciferase gene in the pGL3-enhancer vectors, which were absent of promoter. As shown in Figure 2C, among the vectors of EV-UF, EV-UR, EV-DF, EV-DR, only the vector EV-UF demonstrated luciferase activity higher than pGL3-enhancer vectors. The luciferase activity of EV-UF vectors was increased up to 22 folds compared to that of pGL3-enhancer vectors (Figure 2C), while the luciferase activity of pGL3-basic-derived V-UF vectors were only 3.5 folds higher than pGL3-basic vectors (Figure 2B), suggesting that the fragment was likely a promoter, which was enhanced in EV-UF vectors.

To further exclude the enhancer activity, the fragment -3688/−2680 was inserted into the up or down stream of pGL3-promoter and the vectors of PV-UF, PV-UR, PV-DF and PV-DR were constructed. Both PV-UF and PV-UR did not significantly enhance the activity of pGL3-promoter vector after transfected into HEK-293T cells. Interestingly, PV-DF and PV-DR significantly inhibited the pGL3-promoter activity (Figure 2D), implicating that the fragment might also suppress canonical promoter of *PIWIL2* gene.

Taken together, the results shown above confirm that the fragment -3688/−2680 is truly a promoter specific for *PL2L60* gene, which might serve as a suppressor as well for canonical PIWIL2 promoter.

### Identification of the cis-elements for transcription factors specific for *PL2L60*

**Figure 3:**
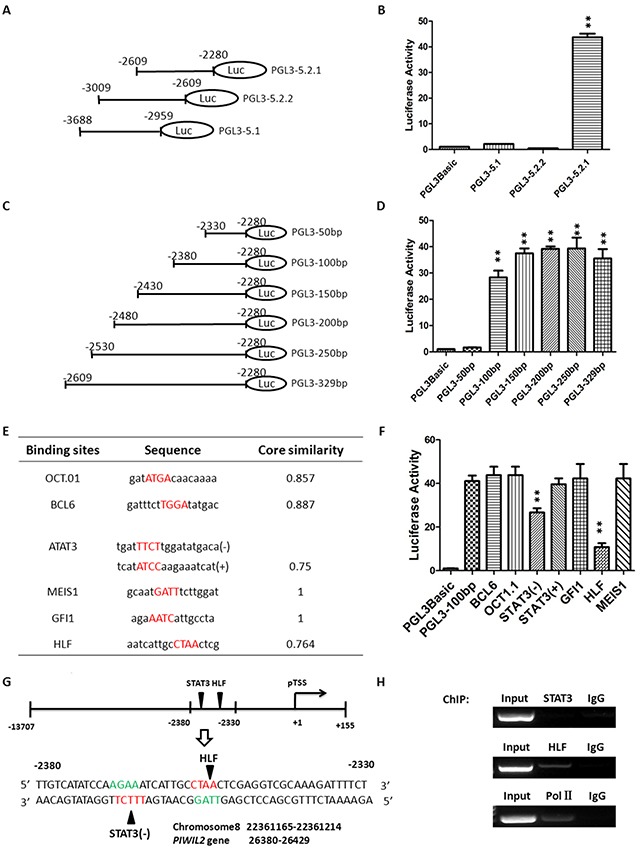
Identification of the potential binding sites for transcription factors in *PL2L60*-specific intragenic promoter **(A)** *Constructs containing truncates of the fragment -3688/−2680*: The region from -3688 to -2680 with promoter activity was truncated into three fragments, which was cloned, respectively, into pGL3-basic report vectors: PGL3-5.2.1 (−2609/−2280), PGL3-5.2.2 (−3009/−2609) and PGL3-5.1 (−3688/−2959). **(B)** *Luciferase activity of the vectors harboring truncates from the fragment -3688/−2680*: HEK-293T cells were transiently co-transfected with 1 μg luciferase report plasmids (PGL3-5.2.1, PGL3-5.2.2 or PGL3-5.1) plus 20 ng expression plasmids for Renilla luciferase (pRL-null), which was used to normalize transfection efficiency. Only does the vector PGL3-5.2.1 (−2609/−2280) exhibit high luciferase activity. **, p < 0.01 as compared to control vectors (PGL3Basic). **(C)** *Serial deletional analysis of the fragment -2609/−2280*: The fragment was sequentially truncated off about 50 nt, six truncates were obtained and each of them was cloned into the pGL3-basic luciferase report vectors. **(D)** *Identification of 50-nt-long truncates with luciferase activity*: The vectors harboring a truncate of interest was transfected, respectively, into HEK-293T cells, and the luciferase activity was measured 48 h later. The fragment -2330/−2280 (PGL-3-50bp) completely lost luciferase activity, suggesting that promoter activity resided in the fragment between -2380 and -2330 (50 nt). **, p < 0.01, as compared to control vectors (PGL3Basic). **(E)** *Bio-informatic prediction of binding sites potentially for transcription factors*: Six potential binding sites for transcription factors in the fragment -2380/−2330 were predicted using Genomatix Software, including OCT1, BCL6, STAT3, MEIS1, GFI1 and HLF. **(F)** *Identification of the specific binding sites responsible for promoter activity by site-direct point mutagenesis*: Site-direct mutagenesis was performed on predicted binding sites for BCL6, OCT1, STAT3(−), STAT3(+), GFI1, HLF and MEIS1, and the mutated fragments (Supplementary Table 7) were cloned into pGL3-basic report vectors, respectively. Each vector harboring a mutated fragment was transfected into HEK-293T cells and luciferase activity was determined. The sites for STAT3 and HLF displayed significantly reduced activity after point mutation, indicating that they were the binding sites for STAT3 and HLF, respectively. **, p < 0.01, as compared to control vectors (PGL3Basic). **(G)** *Positions of binding sites specific for transcription factors STAT3 and HLF in the chromosome and PIWIL2 gene*: Schematic diagram showing the binding sites specific for STAT3 and HLF in the promoter region (−2380/−2330) as well as their position in chromosome 8 (NC_018919.2: 22361165-22361214) and *PIWIL2* gene (26380-26429). **(H)** *Validation of binding sites for STAT3 and HLF in PIWIL2-60-specific promoter*: Chromatin Immunoprecipitation (ChIP) assay followed by DNA PCR were performed to detect the binding sites, respectively, for STAT3, HLF and Pol II in the fragment -2380/−2330. Samples of “input” and “IgG” were used, respectively, as positive and negative controls. Input: samples without treatment with antibody; IgG: samples treated with isotype IgG instead of specific antibody. All the data shown in **B**, **D** and **F** are mean ± SD from 3 independent experiments. Primers used for DNA PCR are shown in Supplementary Table 4.

After defining the active region and confirming promoter property of the putative *PL2L60*-specific promoter, we further determined the binding sites specific for transcription factors (TFs) that transactivate *PL2L60* gene. First, we refined the promoter activity in the region between -3688 and -2680 upstream of the pTSS of *PL2L60*. The fragment -3688/−2680 was further divided into of three fragments, which were amplified by PCR (Supplementary Table 1) and used to construct vectors: PGL3-5.2.1 (−2609/−2280), PGL3-5.2.2 (−3009/−2609) and PGL3-5.1 (−3688/−2959) (Figure 3A). These vectors were transfected into HEK-293T cells. As a result, only did PGL3-5.2.1 (−2609/−2280) retain high luciferase activity (Figure 3B). To further refine the binding region of TFs, a series of deletional analysis was performed with a range of 50 bp difference (Figure 3C). The results showed that the high promoter activity existed in the region between -2380 and -2330 (Figure 3D), suggesting that this region might contain binding sites specific for TFs activating *PL2L60* gene in the host of *PIWIL2*.

Then, we identified the potential binding sites specific for TFs in the region between -2380 and -2330 (Figure 3E). Genomatix software was used to predict binding sites of TFs. Six potential binding sites for transcription factors were identified, which were specific for Octamer (OCT-1), B-cell CLL/lymphoma 6, member B (BCL6), Signal transducer and activator of transcription 3 [STAT3(−) and STAT3(+)], Meis homeobox 1 (MEIS1), Growth factor independence 1 zinc finger protein (GFI1) or Hepatic leukemia factor (HLF) (Figure 3E). To determine which sites were responsible for luciferase activity of the fragment (−2380/−2330), we performed site-directed mutagenesis for each potential binding site (Supplementary Table 2 & 7), constructed corresponding vectors using pGL3-basic report vectors, and transfected them into HEK-293T cells, respectively. As shown in Figure 3F, the luciferase activity was significantly reduced when the putative binding sites specific for STAT3(−) and HLF were mutated, respectively. These two binding sites were closely arranged and only 8 nt away but in the complementary chain of DNA, respectively (Figure 3G). To confirm the binding sites and promoter property, chromatin-immunoprecipitation (ChIP) assay combined with DNA-PCR was performed (Supplementary Table 3). As shown in Figure 3E, the fragment -2380/−2330 was co-precipitated by antibodies to HLF and polymerase II (Pol II), although not by antibody to STAT3 (Figure 3H). However, the fact that both HLF and Pol II recruited to the fragment (−2380/−2330) sufficiently verified the intragenic region acting as a promoter for *PL2L60* gene. Failure to co-precipitate the fragment -2380/−2330 by anti-STAT3 may be associated with STAT3 only binding to STAT3(−) site with low affinity in the complementary chain of the fragment -2380/2330 (Figure 3E, 3G & 3F).

### Activation of intragenic *PL260*-specific promoter by STAT3 and HLF independently

**Figure 4:**
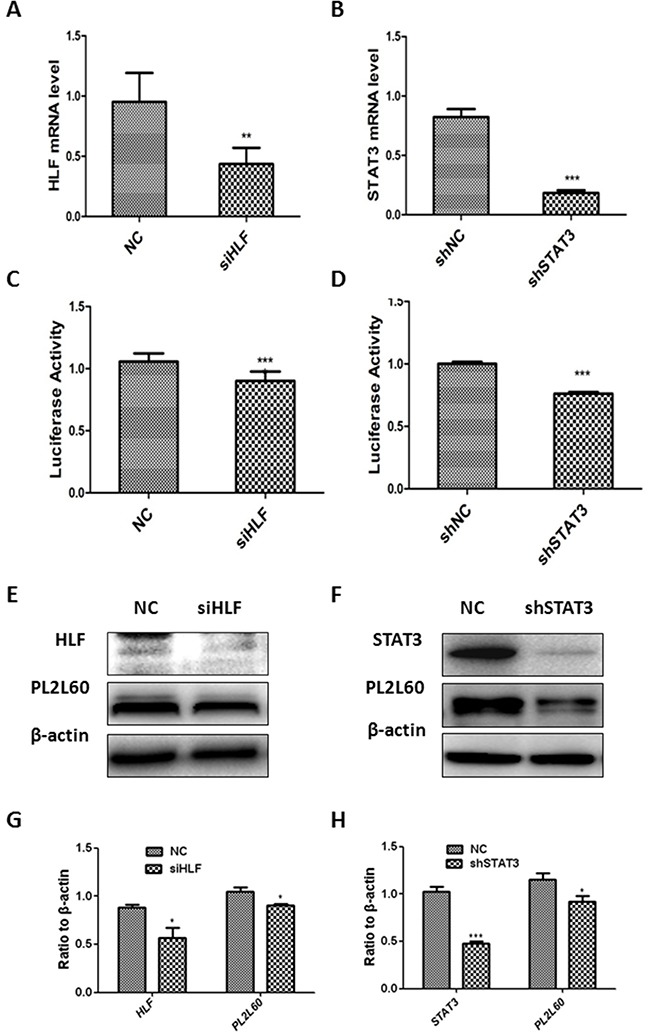
Activation of the *PL2L60*-specific intragenic promoter by STAT3 and HLF **(A, C, E & G)** *Activation of PL2L60-specific intragenic promoter by HLF*: HEK-293T cells were transfected with HLF siRNA or NC siRNA plus vectors PGL3-100bp. The cells were harvested 48 h after transfection and subjected to real-time PCR analyses of *PL2L60* mRNAs **(A)**, leuciferase activity assays for *PL2L60*-specific intragenic promoter activity **(C)** and Western blotting analyses of HLF and PL2L60 expressions **(E & G)**. **(B, D, F & H)** *Activation of PL2L60-specific promoter by STAT3*: HEK-293T cells were infected with lentiviral STAT3 shRNAs or lentiviral NC shRNAs. Once stable shSTAT3 and shNC cell lines had been established, they were transfected with PGL-3 100bp plasmids for 48 hrs and subjected to analysis of STAT3 mRNAs **(B)**, *PL2L60*-specific promoter activity **(D)** and protein expressions of STAT3 and PL2L60 **(F & H)** as described above. The data shown are mean ± SE from three independent experiments **(A, B, C, D, G & H)** and a representative from at least three independent experiments **(E & F)**. siHLF: HLF siRNAs; NC: NC siRNAs; shSTAT3: stable cell lines expressing STAT3 shRNAs; shNC: stable cell lines expressing mock (NC) shRNAs. *, p < 0.05; **, p < 0.01; ***, p < 0.001, when compared to control groups.

To determine whether the intragenic *PL2L60*-specific promoter was activated coordinately by STAT3 and HLF, we knocked down STAT3 or HLF mRNAs in HEK-293T cells using RNA interference techniques. The HEK-293T cells were transfected with STAT3 shRNAs (forming a stable shSTAT3 cell line) and HLF siRNAs, respectively, and simultaneously transfected with pGL3-basic reporter vectors harboring the fragment -2380/−2330. As a result, the mRNAs (Figure 4A & 4B), which were assessed with real-time PCR (Supplementary Table 4), and proteins (Figure 4E/4G & 4F/4H) of both STAT3 and HLF assessed with Western blot were significantly decreased. In line with the results, the luciferase activity was also synchronously reduced in the HLF siRNA-treated HEK-293T cells (Figure 4C) and shSTAT3 cell lines (Figure 4D). The results suggest that the decreased mRNAs and proteins of STAT3 and HLF, respectively, led to the reduced activation of the intragenic *PL2L60*-specifc promoter. This observation was further confirmed by the reduced expression of PL2L60 proteins in the HLF siRNA-treated cells (Figure 4E & 4G) and shSTAT3 cell lines (Figure 4F & 4H). These results confirmed that both HLF and STAT3 could stimulate the expressions of PL2L60 proteins through activating intragenic *PL2L60*-specific promoter in the host gene of *PIWIL2*. Interestingly, HLF and STAT3 might transactivate *PL2L60*-specific promoter interdependently, because co-transfection of shSTAT3 cell lines with HLF siRNAs and pGL3-basic vectors harboring the fragment -2380/−2330 had no additive effects on the luciferase activity and did not further reduce PL2L60 proteins expression (not shown). This may be related to the steric interaction between HLF and STAT3, which was required for transactivation of *PL2L60*, because the cis-acting elements for STAT3 and HLF were located in two complementary DNA chains, respectively, but closely rearranged (Figure 3G). In this situation, STAT3 might not tightly bind its cis-acting element and be released from nucleotides in ChIP assay. Overall, the results confirm that the *PL2L60*-specific intragenic promoter in the host gene *PIWIL2* may be activated by HLF and STAT3 interdependently, i.e. through a steric interaction.

### Enhanced activation of *PL2L60*-specific intragenic promoters in the *Piwil2*-defective *milie*u

**Figure 5:**
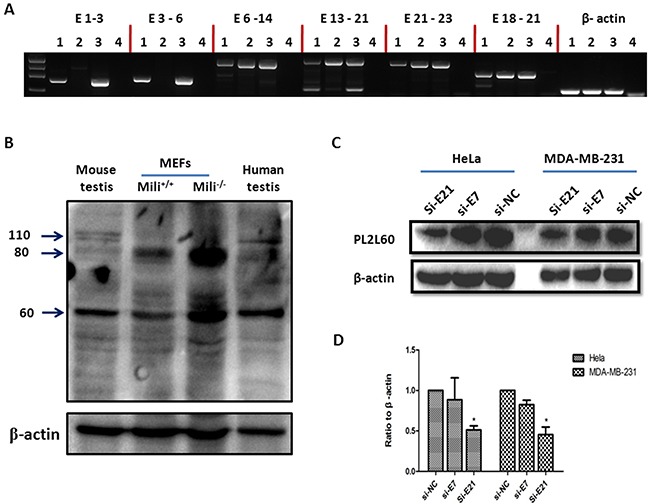
Aberrant activation of *PIWIL2* gene by intragenic promoters independently of the integrity of host gene **(A)** *Expression of short form mRMAs of Piwil2 in the primary mili^−/−^ MEFs*: RNAs were extracted from the testicular tissues and MEFs of wild-type (wt) or *Piwil2*-deficient (*mili*^−/−^) mice [5] and subjected to GEM RT-PCR analysis for the length of Piwil2 mRNAs [1]. As results, *mili*^−/−^ MEFs expressed a short form mRNAs covering from exons 6 to exon 23, whereas the testicular tissues and MEFs from wt mice expressed full length of Piwil2 mRNAs, suggesting that intragenic promoter activation is independent of the integrity of host gene. Lane 1: wt MEF, Lane 2: *mili*^−/−^ MEF; Lane 3: wt testicular tissues and Lane 4: H_2_O. The primer pairs specific for E1-3, E3-6, E6-14, E13-21 and E21-23 of Piwil2 were used for GEM RT-PCR analysis, and the primer pair specific for E18-21 was used as a positive control (Supplementary Table 5). **(B)** *Piwil2 and PL2L proteins expression in Piwil2-deficient MEFs*: Primary *mili*^−/−^ MEFs, wt MEFs (both were at 30 generations of *in vitro* culture), and testicular tissues from a wt mouse and a patient were analyzed for the expressions of Piwil2 and PL2L proteins by Western blot, using a mouse mAb to a peptide common for Piwil2 and PL2L of both mouse and human (clone Kao3 [1]). PL2L60 and PL2L80 but not Piwil2 were detected in *mili*^−/−^ MEFs. As comparisons, PL2L60, PL2L80 and Piwil2 were detected in wt MEFs, and testicular tissues of human and mice. However, PL2L60 and PL2L80 were also detected in *mili*^−/−^ MEFs in a much higher level than in wt MEFs and testicular tissues. The detection of a high level of PL2L80 reflected potentially a *PIWIL2-80*-specific intragenic promoter that was activated more strongly in *mili*^−/−^ MEFs than in wt MEFs. **(C & D)** *PL2L60 protein expression in E21-specific siRNA-treated tumor cell lines*: Human cancer cell lines of breast (MDA-MB-231) and cervix (HeLa) were treated with siE21, siE7 and siNC for 72 h and analyzed for PL2L60 expression by Western blot. Top panel: a representative result of Western blot, using polyclonal rabbit antibody to PL2L peptides with the same specificity as mAb Kao3 [1]; bottom panel: a summary of three independent experiments (mean ± SD). siE7: siRNA targeting exon 7 of PIWIL2 transcripts; siE21: siRNAs targeting exon 21 of PIWIL2 transcripts; and siNC: scramble siRNAs used as a negative control. *, p < 0.05, compared to siE7 or siNC.

To determine whether activation of intragenic promoters was dependent on the integrity of host gene, we examined transcriptional expression of *Piwil2* in the *mili*^−/−^ (*Piwil2*^−/−^) murine embryo fibroblasts (MEFs), using Gene-Exon-Mapping Reverse transcription polymerase chain reaction (GEM RT-PCR) assay [1]. A serial of tiling primers covering full length Piwil2 transcripts were used for GEM RT-PCR assays (Supplementary Table 5). As shown in Figure 5A, the *mili*^−/−^ MEFs, in which the *Piwil2* gene was genetically disrupted via homologous recombination targeting between the exon 2 and exon 5 [5], did not completely abrogate *Piwil2* gene expression. Although full length Piwil2 transcripts were not detected, the transcripts truncated at 5′-end spanning from exon 6 to exon 23 were detectable. The truncated transcripts were absent of exons 1 - 5. As controls, full length Piwil2 transcripts (exons 1-23) were detected in the wild type (wt) MEFs and testicular cells of wt mice (Figure 5A). However, the truncated transcripts was longer than what we expected for PL2L60 transcripts spanning from exons 11 to 23, suggesting that in addition to *PL2L60*-specific intragenic promoter, another intragenic promoter might exist in the upstream of exon 5, and its activation might result in PL2L80 or PL2L80A expression [1, 27]. Consistently with the hypothesis, two PL2L proteins bands with molecular weight (MW) about 80 and 60 kDa were identified by Western blot in the testicular cells of human and *mili*^−/−^ mice as well as in the *mili*^−/−^ and WT MEFs (Figure 5B). Importantly, PL2L60 and probably PL2L80 or PL2L80A [1, 27] were remarkably up regulated in *mili*^−/−^ MEFs, as compared to those in wt MEFs as well as in the testicular cells of human and wt mice (Figure 5B). As we had expected [1, 2, 13], the Piwil2 proteins (110 kDa) were detected in the testicular cells of human and mice as well as in the mili^+/+^ MEF, although very low in level, but not in the mili^−/−^ MEF (Figure 5B). The results supported the notion that in addition to the *PL2L60*-specific intragenic promoter, a *PL2L80*-specific intragenic promoter might exist in the intron between the exon 5 and exon 6, which may transcribe *PL2L8*0 mRNAs [1, 27]. These results suggest that aberrant activation of *PIWIL2* gene by intragenic promoters was dependent on cellular *milieu* independently of the integrity of host gene.

To further verify the existence of PL2L60 mRNAs (amRNA of *PIWIL2*), we examined the expression of PL2L60 proteins after knockdown of putative PL2L60 mRNAs in human tumor cell lines, using the siE21 siRNAs, which targeted exon 21 of *PIWIL2*, PL2L80 and PL2L60 transcripts. On the other hand, to verify the mRNAs knocked down by siE21 were mainly *PL2L60*-specific, we used siE7 siRNAs, which targeted exon 7 of *PIWIL2* and PL2L80 transcripts but had no effect on PL2L60 transcripts, and siNC (negative control siRNA) as controls (Supplementary Table 6). As expected, knocking down of mRNAs using siE21 significantly inhibited the expression of PL2L60 proteins by about 45% and 50% in the tumor cells of cervix and breast, respectively. In contrast, siE7, which was effective in knocking down full length PIWIL2 mRNAs and PL2L80 mRNAs (not shown), and siNC had no significant effects on PL2L60 protein expression (Figure 5C & 5D). Again, these results verified that the PL2L60 proteins were translated from PL2L60 mRNAs. It should be noted that little full length of Piwil2 proteins were detected in wt MEFs (Figure 5B) and human tumor cell lines (not shown) [1], this is consistent with our previous observations that full length PIWIL2 proteins were expressed in a lower level in normal somatic cells and tumor cell lines, while PL2L60 proteins were predominantly expressed in tumor cell lines [1, 13].

Taken together, these results suggest that activation of intragenic *PL2L60*-specific promoter is not dependent on the integrity of host gene *PIWIL*2, but may be enhanced in the *PIWIL2*-defective *milieu*.

## DISCUSSION

In addition to gametogenesis [2, 5, 40], *PIWIL2* can promote tumorigenesis through upregulating several signal transduction pathways [1, 2, 10, 41–46] and inhibiting apoptotic death of tumor cells via activation of Stat3/Bcl-XL pathway [10, 11]. However, these functions of PIWIL2 in tumor development remain controversial, because most of the commercial available antibodies specific for PIWIL2 could not distinguish the full length PIWIL2 from its variants [1, 2]. In primary breast and cervical cancers, full length PIWIL2 proteins were detected mainly in apoptotic tumor cells but little in the living tumor cells, when monoclonal antibody (mAb) to PIWIL2 was used. In contrast, PIWIL2 variants PL2L proteins, such as PL2L60, were detected abundantly in various types of tumor tissues and tumor cell lines [1, 27], suggesting that the tumorigenic functions of *PIWIL2* might be mediated mainly by *PIWIL2* variants [1, 2, 17, 28]. However, the mechanisms by which PL2L60 promotes tumorigenesis remain to be elucidated [1, 2, 8, 13, 17, 28].

In the present study, we have cloned and characterized a 50-bp-long element with promoter activity in the intron 10, which locates between exon 10 and 11 of *PIWIL2* gene (in the genome: Homo sapiens chromosome 8, alternate assembly CHM1_1.1: 22334485- 22415561; in the *PIWIL2* gene: 26380-26429; Figure 1). This promoter is functional in transcribing PL2L60 mRNAs, because both luciferase activity of the promoter and the levels of PL2L60 proteins were synchronously decreased when the STAT3 and HLF, two activators specific for the intragenic promoter, were knocked down, respectively, by their specific siRNAs (Figure 4). In addition, Pol II, a hallmark of active promoter, was detected in this region by ChIP analysis (Figure 3H). Finally, siE21 rather than siE7 specifically knocked down PL2L60 mRNAs (Figure 5C & 5D). These results verify that PL2L60 proteins in tumor cell lines [1, 2] are resulted from the activation of intragenic *PL2L60*-specific promoter in the host gene of *PIWIL2*.

To refine the *PL2L60*-specific intragenic promoter, a series of pGL3-basic report vectors were constructed with fragments from a 13,862 bp fragment (−13707/+155) surrounding the pTSS of the putative *PL2L60*–specific promoter [1]. The promoter activity was detected in a subfragment -3688 to-2280. To further verify the promoter activity and exclude the intragenic enhancer activity of the fragment [39], pGL3-basic, pGL3-enhancer and pGL3-promoter vectors were used to test the promoter activity and enhancer activity of the fragment -3688/−2880, respectively. The promoter activity of the fragment was confirmed and the enhancer activity was exculed completely, because the luciferase activity was detected only when the fragment was inserted into the upstream of the luciferase gene of pGL3-basic (V-UF) or pGL3-enhancer (EV-UF) in 5′- 3′ forward direction (Figure 2B & 2C), and no enhanced activity was observed when the fragment was inserted into pGL3-promoter vector (PV-UF) (Figure 2C).

To determine the cis-acting and trans-acting elements of the promoter, a number of deletional mutants were generated from the region of -3688/−2280, from which only 50 nt in the region of -2380/−2330 responsible for promoter activity were identified. In this 50-nt-long fragment, six potential cis-acting elements were predicted, and two of them were verified by site-directed mutagenesis as cis-elements specific for transcription factors (TFs) HLF and STAT3, respectively (Figure 3F).

The promoter activity for *PL2L60* was further verified by the specific recruitments of HLF and Pol II to the region, although STAT3 binding to its cis-element was not confirmed by ChIP assay. It is likely that the STAT3 has lower affinity for its cis-elements than HLF and fails to be precipitated by anti-STAT3. However, knockdown of STAT3 significantly reduced both luciferase activity of the promoter and PL2L60 protein expression (Figure 4B, 4D, 4F & 4H), similarly to knocking down of HLF (Figure 4A, 4C, 4E & 4G). Therefore, the promoter activity of fragment -2380/−2330 is *PL2L60*-specific.

Interestingly, co-knockdown of STAT3 and HLF using STAT3 shRNAs plus HLF siRNAs did not result in an additive effect on both luciferase activity of the promoter and PL2L60 protein expression (not shown). The phenomenon suggests that HLF and STAT3 might interdependently transactivate *PL2L60*-specific promoter through a mechanism of steric interaction. The hypothesis is at least partially supported by the fact that the binding sites for STAT3 and HLF were closely arranged but in two complementary DNA chains, respectively (Figure 3G), providing a condition for steric interaction between the HLF and STAT3. This is consistent with the notion that *PL2L60* is not activated normally in the somatic cells but might be more tightly regulated by transcription factors [1, 8]. It would be highly appreciated for complete understanding of the mechanisms underlying the steric interaction between STAT3 and HLF required for transactivation of *PL2L60*-specific intragenic promoter.

Although we could not firmly conclude that STAT3 be a transcription factor specific for *PL2L60* gene at present study because of a lack of positive result from ChIP analysis, the regulatory function of STAT3 on *PL2L60* promoter is highly appreciated. It has been demonstrated that the over-expression of PL2L60 in cancer cells is associated with up-regulation of STAT3 mRNAs [1]. The *STAT3* has been considered to play a critical role in cell differentiation, survival, proliferation, anti-apoptosis and angiogenesis [47–49]. In particular, its expression is closely associated with the metastasis and invasiveness of cancers [50–52]. Constitutive over-activation of *STAT3* [50, 52–55] and constitutive expression of PL2L60 [1] have been demonstrated in various types of human cancers. In this study, STAT3 is found to activate *PL2L60*-specific promoter, which is associated with increased expression of PL2L60 (Figure 4D, 4F & 4H) and, in turn, PL2L60 may stimulate *STAT3* gene expression in tumor cells [1], suggesting that a feedback pathway of STAT3/PL2L60 for promoting tumorigenesis might exist. It is worthwhile to further elucidate the interactive mechanisms between STAT3 and PL2L60.

In addition to STAT3, HLF is also found to stimulate *PL2L60* transcription in HEK-293T cells. The function of *HLF* has not yet been elucidated in tumorigenesis, although it appears to play important roles [56]. HLF belongs to a member of the proline and acidic-rich (PAR) protein family, a subset of the bZIP transcription factors [57]. It forms homodimers or heterodimers with other PAR family members through binding sequence-specific promoter elements to activate transcription. HLF expression certainly cycles with circadian rhythms and is universally accepted as an output mediator of this process [58]. In acute B-lineage leukemia, *HLF* gene translocating to fuse with the *E2A* gene drives tumor development [59, 60]. It is interesting to investigate whether the HLF-E2A is associated with PL2L60 expression in tumors.

We have previously reported that disrupting *Piwil2* via homologous recombination targeting from exon 2 to exon 5 did not completely eliminate *Piwil2* gene in the testicular cells, because the transcripts spanning exons from 6 to 23 of *PIWIL2* were detected by GEM RT-PCR, and the proteins PL2L80 and PL2L60 were detected by Western blot [1]. In this study, we examined the transcripts and proteins in the mili^−/−^ MEFs and observed the same results (Figure 5A & 5B) as observed in mili^−/−^ testicular tissues [1]. The observations strongly suggest that in addition to *PL2L60*-specific intragenic promoter, a *PL2L80*-specific intragenic promoter might exist for upregulating PL2L80 or PL2L80A expression [1, 27]. Moreover, the levels both PL2L80 and PL2L60 proteins in mili^−/−^ MEFs were much higher than in mili^+/+^ MEFs (Figure 5B), suggesting that activation of the intragenic promoter is independent of the integrity of host gene, and even the activation be enhanced when the host gene is disrupted (Figure 5B). This is of significance especially in the status of cancer gene mutations, which may result in aberrant activation of the host genes, leading to tumorigenesis. This might be related to carcinogenic transformation of mili^−/−^ MEFs, because we have observed that the mili^−/−^ MEFs acquired tumorigenic capacity after long-term *in vitro* culture (unpublished data).

**Figure 6:**
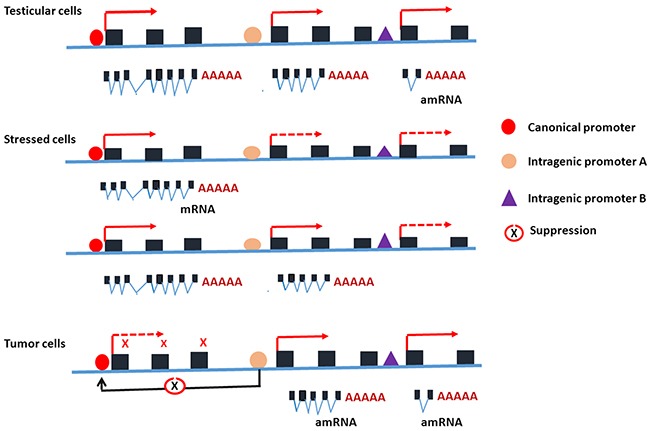
Schematic depiction for the potential mechanisms underlying the activation of intragenic promoter in various conditions and its relationship to the canonical promoter of the host gene *PIWIL2* In the testicular cells, both the canonical promoter and intragenic promoters of *PIWIL2* are activated during gametogenesis and the proteins of PIWILL2, PL2L80 and PL2L60 are expressed [1, 27]. In the somatic cells, the canonical promoter and intragenic promoters of *PIWIL2* are silent, but activated when the cells become stressed after exposure to environmental agents including DNA-damage-inducing agents or carcinogens [13]. The canonical promoter of *PIWIL2* may be activated transiently in responding to stresses, serving as a tumor barrier gene [2, 13], while the activation of its intragenic promoters may lead to cell transformation and tumor initiation [9, 17, 28]. In the tumor cells, the canonical promoter of *PIWIL2* is usually silent, while intragenic promoters are activated to promote tumorigenesis [1, 13], probably through inhibition of canonical promoter of *PIWIL2*. However, *PIWIL2* could be activated in the tumor cells under the stressed condition such as hypoxia. Solid line arrows: activated; dashed line arrows: silent; and black line arrows: inhibited.

The discovery of intragenic promoters in the *PIWIL2* gene reveals a novel, important mechanism by which PIWIL2 plays differential roles in the development and tumorigenesis [1, 2, 27]. Intragenic promoter has been described in tRNA transcription, open reading frame (ORF) or exons as an encrypted promoter and intragenic enhancers acting as alternative promoters [39, 61]. Identification of the *PL2L60*-specific intragenic promoter reveals that (a) activation of an intragenic promoter may be associated with aberrant activation of a host gene, resulting in a protein product with the function being opposite to that of host gene; (b) intragenic promoter may be activated by multiple specific TFs through steric interaction; (c) intragenic promoters are usually inactive in somatic cells under physiological conditions but may be over-activated by pathological stimuli, such as stress and carcinogenic agents; (d) the relationship between canonical and intragenic promoters might be mutually regulated, because pGL3-promoter activity was significantly inhibited when *PL2L60*-specific promoter was inserted into downstream of the promoter in either directions (Figure 2D); (e) traditional RT-PCR technique is not sufficient to detect the mRNAs reflecting the status of gene activation, especially for the genes that are disrupted and associated with tumorigenesis. The potential mechanisms of intragenic promoter activation and its relationship to the host gene activation under various conditions are schematically depicted in Figure 6.

Recently, it has been reported that intragenic enhancer can also act as an alternative promoter to transcribe meRNA [39], although the function of meRNA remained elusive. In this study, we have revealed that the *PL2L60*-specific intragenic promoter is distinct from the intragenic enhancer as well as canonical promoter. The finding is supported by the following additional evidences: first, the *PL2L60*-specific intragenic promoter was activated in both pGL3-basic and pGL3-enhancer luciferase report vectors unidirectionally but not in the pGL3-promoter vector (Figure 2); secondly, the binding sites of *PL2L60*-specific promoter for TFs is located in the intron away from pTSS about 2330 nt, distinct from canonical promoter, which usually located about 50 – 100 nt away from pTSS. Therefore, *PL2L60*-specific intragenic promoter is a novel class of regulatory elements in genes, signifying the importance for the development and tumorigenesis.

The transcripts resulting from intragenic promoter activation do encode proteins with altered function; we call this class of mRNAs as aberrant mRNA (amRNA) to distinguish the mRNAs transcribed by canonical promoters or meRNAs transcribed by enhancer-derived alternative intragenic promoters. The protein products of amRNA are opposite to the proteins products of host gene's mRNAs. For an example, the host gene *PIWIL2* is characterized by conserved PAZ domain and PIWI domain [2], however, its intragenic *PL2L60*-specific promoter can transcribe mRNAs that may give rise to the proteins lacking PAZ domain, resulting in opposite function to PIWIL2 [1].

In summary, we have revealed a mechanism underlying the aberrant activation of *PIWIL2* gene in somatic cells. A *PL2L60*-specific promoter within the *PIWIL2* gene has been identified, cloned and characterized, which is located between exon 10 and 11 of *PIWIL2* gene and can transcribe amRNA, distinct from the recently reported meRNA transcribed by an intragenic enhancer-derived alternative promoter. The intragenic *PL2L60*-specific promoter can be activated by HLF and, likely, STAT3, probably through the interdependent steric interaction. Its activation is not dependent on the integrity of host gene but may be enhanced by mutations of host genes. Our study has confirmed that the intragenic promoter may contribute to aberrant activation of a host gene, a potentially important mechanism for activation of a cancer gene in tumorigenesis. The finding gives rise to a novel insight into the mechanisms mediating cancer development as well as into therapeutic strategies for cancers.

## EXPERIMENTAL PROCEDURES

### Ethics statement

One sample of testicular parenchyma (normal testis) was obtained from a patient with prostate cancer underwent orchiectomy at the Department of Urology, Renji Hospital, with the permission of Institutional Review Board of Renji Hospital. Animal experiments were conducted based on Animal Use Protocols approved by the Institutional Animal Care and Use Committee (IACUC), Renji Hospital.

### Animals, cell lines and cell culture

Male C57BL/B6 (B6) mice and mili^−/−^ B6 mice were used at the age of 8 −12 wk. The mili^−/−^ mice, which were obtained from Dr. Haifan Lin's laboratory [5], were bred and maintained in the animal pathogen-free facility at the Renji Hospital. Human cell lines used in experiments included the HEK-293T (ATCC CRL-3216, embryonic kidney), MDA-MB-231 (ATCC HTB 26, mammary gland/breast; derived from metastatic site: pleural effusion) and HeLa cells (ATCC CCL-2, cervical adenocarcinoma). Murine cell lines included the mili^−/−^ MEF (mouse embryonic fibroblasts) and mili^+/+^ MEFs, which were established in our laboratory [1]. All the cell lines used in this study were grown in Dulbecco's modified Eagle medium (Invitrogen) containing 10% fetal bovine serum (Invitrogen) supplemented with 0.1mg/ml Penicillin-Streptomycin (D10F) and maintained at 37oC in a humidified atmosphere with 5% CO_2_ and 95% air.

### Construction of vectors with the fragments segmented from putative *PL2L60*-specifc intragenic promoter

Six pairs of primers that contain restriction sites were synthesized for amplifying six putative *PL2L60*-specifc intragenic promoter fragments, covering nucleotide sequence from +155 to −13707 (Supplementary Table 1). All the primers used for PCR were purchased from Sanggon Biotech (Shanghai, China). *PL2L60*-specifc promoter fragments including -2409/+155, -3688/−2280, -6228/−3633, -8673/−6193, -11225/−8643 and -13707/−11190 were amplified by PCR from human genomic DNA. The fragments were cloned into pGL3-basic luciferase reporter vectors (Promega, USA), upstream of the reporter firefly (*Photinus pyralis*) luciferase gene. Luciferase activity was determined from the fragments by transfection of the vectors into HEK-293T cells, using jetPRIME® transfection reagent (Polyplus-transfection SA), according to manufacturer's instruction. Biological and technical triplicates were used to ensure reproducibility. Cells were lyzed 48 h after the transfection and the activity of both firefly and *Renilla reniformis* luciferases was assessed using Dual Luciferase Reporter Assay System (Promega, USA) and Tecan GENios Pro Luminometer (MTX Lab Systems, USA), according to the manufacturers’ instructions.

To further refine the regions with promoter activity, vectors of PGL3-5.1, PGL3-5.2.1 and PGL3-5.2.2 were constructed with three subfragments of 729bp, 329bp and 400bp, respectively, which were derived from a promoter-containing fragment. The vector PGL3-5.2.1 showing promoter activity was further used as template to serially construct a number of vectors with 50 bp or more increments (PGL3-50bp, PGL3-100bp, PGL3-150bp, PGL3-200bp, PGL3-250bp and PGL3-329bp). These vectors were transfected into HEK-293T cells to screen promoter activity within a 50-nt fragment.

In the experiments described above, each fragment was purified using the QIAquick Gel Extraction Kit (Qiagen, USA) according to the manufacturer's recommendations and sub-cloned into the pGL3-basic luciferase reporter vector (Promega, USA). All constructs were verified by DNA sequencing. All the primers used for amplifying the fragments are shown in Supplementary Table 1.

### Promoter validation and enhancer activity assay

To distinguish whether the activity of an identified DNA fragment was associated with a promoter or an enhancer, a serial of vectors was constructed with a fragment (−3688/−2280) showing high luciferase activity. The fragment was cloned into pGL3-basic vectors or pGL3-enhancer vectors up and down stream of the luciferase gene as well as in forward and reverse direction, respectively, for validation of promoter activity. On the other hand, the fragment was cloned into pGL3-promoter vectors in the same way as above to exclude enhancer activity of the fragment. The vectors transfected into HEK-293T cells for luciferase activity assay as described above.

### Transient transfection and luciferase reporter assay

HEK-293T Cells were plated into 6-well plates (2 × 10^5^ cells/well) and co-transfected with 1 μg of Firefly luciferase reporter vectors of interest and 20 ng of the Renilla luciferase expression vectors (pRL-TK), using jetPRIME® transfection reagent (Polyplus-transfection SA) 24 h later. After 48 h of transfection, the cells were lysed with passive lysis buffer (Promega, USA), and Luciferase activity was determined in cell extracts using the Dual-Luciferase Reporter Assay kit (Promega, USA). Data were expressed as the ratio of Firefly to Renilla luciferase activities.

### Site-directed mutagenesis

PGL3-100bp vectors were used as the template for site-directed mutagenesis. The PCR cycling parameters were used as follows: 1 cycle of 98°C for 30 s, 35 cycles of 98°C for 10 s, 60°C for 30s, and 72°C for 2 min, 1 cycle of 72°C for 7 min for point mutation. The primers are shown in Supplementary Table 2. The sequence of inserts was verified by DNA sequence analysis.

### Real-time polymerase chain reaction (PCR) and touch-down reverse transcription (RT)-PCR

Total cellular RNAs were extracted with Trizol reagent (Invitrogen). For real-time PCR, a total of 500 ng of RNAs was used for reverse transcription with the Thermo Scientific Revert Aid First Strand cDNA Synthesis Kit (Thermo). SYBR Green PCR real-time PCR Master Mix (Toyobo, Osaka, Japan) was used according to the manufacturer's instructions. First, the reaction was incubated at 95°C for 10 min, then 40 cycles of 95°C for 15 s and 60°C for 1 min, followed by 95°C for 15 s. The relative changes in mRNAs levels were normalized for β-actin mRNAs in the same samples. The primers used are summarized in Supplementary Table 3.

For RT-PCR, total RNA was extracted from cell lines or *de novo* isolated testicular cells and splenocytes, as previously described [1, 8]. Briefly, the cDNA was generated by reverse transcription using Superscript II (Invitrogen, CA) and oligo (dT) in a 20 μl reaction containing 1 μg of total RNA, which was pretreated with RNase-free DNase I (Invitrogen, CA) to eliminate contaminating genomic DNA. Then, an aliquot of 0.5 μl cDNA was used in each 20 μl PCR reaction, using PCR Master Mix (Promega, Ca). The following conditions were used for 35 cycles of PCR: an initial denaturation at 95°C for 5 min followed by denaturation at 94°C for 30 seconds, annealing at 65°C for 1 min, touchdown −1°C per cycle, and extension at 72°C for 1 min for a total of 10 cycles. Then the condition was fixed for 25 cycles of denaturation at 94°C for 30 seconds, annealing at 50°C for 1 min, and extension at 72°C for 1 min with a final extension at 72°C for 10 min. PCR products were analyzed by 1.5% agarose gel. The sequence of primers is listed in the Supplementary Table 5.

### Gene-Exon-Mapping reverse transcription polymerase chain reaction (GEM RT-PCR)

To identify Piwil2 variants, GEM RT-PCR was performed as previously described [1]. Five tiling primer pairs specific for murine *Piwil2* were designed (Supplementary Table 5), which cover the entire Piwil2 transcripts. Each primer pair spanned at least one intron based on the databases of Ensembl (http://www.ensembl.org). The upstream of each pair of primers overlapped the downstream of previous one. PCR was performed as described previously [1].

### RNA interference (RNAi) technique

Pre-experimentally verified small interference RNAs (siRNAs) for STAT3, HLF, Exon 7 of human *PIWIL2* (siE7), Exon 21 of human *PIWIL2* (siE21) and negative control (NC) were purchased from Shanghai Gene Pharma. Transient transfections were carried out according to the manufacturer's instructions by using jetPRIME® transfection reagent (Polyplus-transfection SA). HEK-293T cells were plated into 6-well plates at 2 × 10^5^ cells/well for 24 h prior to treatment with siRNAs (Supplementary Table 6). Transfected cells were harvested 48 h after transfection and subjected for Western blot analysis.

### Western blot analysis

As previously described with modification [1], the cells and tissues were collected and lysed in Radio-Immunoprecipitation Assay (RIPA) buffer (Thermo Fisher, Rockford, IL, USA), containing 1% phenylmethylsulfonyl fluoride (PMSF). Protein concentration was quantified with the BCA Protein Kit (Beyotime, China). Lysates (10 μg proteins) were separated by 10% SDS-PAGE gels and transferred to a PVDF membrane (Bio-Rad, Hercules, CA, USA). The membranes were blocked with 5% Bovine Serum Albumin in TBS/Tween20 (TBST) for 2 hours at room temperature, and then probed with primary antibodies against proteins of interest overnight at 4°C. After incubated with secondary antibodies for 1 hour at room temperature, protein signal was detected with the ECL chemiluminescent detection system (Bio-Rad), and protein levels were normalized to β-actin.

### Chromatin immunoprecipitation (ChIP) assay

The EZ-ChIP ^TM^Chromatin Immunoprecipitation Kit (Millipore, USA) was used for the ChIP assay. Briefly, 5 × 10^6^ cells were fixed with 1% formaldehyde in PBS for 10 min to cross-link DNA with associated proteins and the cells were then washed and harvested in PBS. The pelleted cells were lysed on ice in SDS lysis buffer and then sonicated on ice for 10 seconds, 10 times (on time, 10 s; off time, 59s). The sheared DNA was incubated with normal rabbit IgG (Cat. # 2729; Cell Signaling Inc.) as a negative control, Polymerase II (Pol II) antibodies (H-224;sc-9001; Santa Cruz Biotechnology, Santa Cruz, CA) as a positive control, and human STAT3 antibodies (Cat. #4904S; Cell Signaling Inc.), or human HLF antibodies (H-71; sc-367607; Santa Cruz Biotechnology, Santa Cruz, CA) overnight at 4°C, followed by 1 h of incubation with DNA/protein A-agarose beads. 10% of the sample was kept as input. Protein A-agarose beads pellets were sequentially washed with a low salt buffer, a high salt buffer, a LiCl washing buffer, and TE buffer. Protein-DNA complexes were eluted in a buffer containing 1% SDS and 0.1 M NaHCO3. Cross-linking was reversed with 200 mM NaCl overnight at 65°C, followed by incubation in a buffer containing 40 mM Tris-HCl, pH 6.5, 10 mM EDTA, and 20 μg of proteinase K for 2h at 55°C. DNA was purified with the TIANquick Midi Purification Kit (TIANGEN, China). Prepared samples were amplified using specifically designed ChIP primers for DNA-PCR covering the region of interest (Supplementary Table 4). The DNA PCR conditions were as follows: 1 cycle of 94°C for 3 min, 34 cycles of 94°C for 20 s, 59°C for30 s and 72°C for 30 s, 72°C for 2 min, and the products were storage at -20°C for latter assay.

### Statistical analysis

Results were expressed as the mean ± SE of at least three independent experiments and analyzed by ANOVA using GraphPad software. The p value was determined by Student-T test. A p value < 0.05 (*) was considered statistically significant, and p < 0.01 (**) or < 0.001 (***) was considered as highly significant.

## SUPPLEMENTARY TABLES





## References

[1] Ye Y, Yin DT, Chen L, Zhou Q, Shen R, He G, Yan Q, Tong Z, Issekutz AC, Shapiro CL, Barsky SH, Lin H, Li JJ, Gao JX (2010). Identification of Piwil2-like (PL2L) proteins that promote tumorigenesis. PLoS ONE.

[2] Gao JX, Liu N, Wu HL (2014). PIWIL2 (piwi-like RNA-mediated gene silencing 2). Atlas Genet Cytogenet Oncol Haematol.

[3] Lin H, Spradling AC (1997). A novel group of pumilio mutations affects the asymmetric division of germline stem cells in the Drosophila ovary. Development.

[4] Kuramochi-Miyagawa S, Kimura T, Yomogida K, Kuroiwa A, Tadokoro Y, Fujita Y, Sato M, Matsuda Y, Nakano T (2001). Two mouse piwi-related genes: miwi and mili. Mech Dev.

[5] Kuramochi-Miyagawa S, Kimura T, Ijiri TW, Isobe T, Asada N, Fujita Y, Ikawa M, Iwai N, Okabe M, Deng W, Lin H, Matsuda Y, Nakano T (2004). Mili, a mammalian member of piwi family gene, is essential for spermatogenesis. Development.

[6] Sasaki T, Shiohama A, Minoshima S, Shimizu N (2003). Identification of eight members of the Argonaute family in the human genome small star, filled. Genomics.

[7] Lee JH, Engel W, Nayernia K (2006). Stem cell protein Piwil2 modulates expression of murine spermatogonial stem cell expressed genes. Mol Reprod Dev.

[8] Chen L, Shen R, Ye Y, Pu XA, Liu X, Duan W, Wen J, Zimmerer J, Wang Y, Liu Y, Lasky LC, Heerema NA, Perrotti D (2007). Precancerous stem cells have the potential for both benign and malignant differentiation. PLoS One.

[9] Gao JX (2008). Cancer stem cells: the lessons from precancerous stem cells. J Cell Mol Med.

[10] Lee JH, Jung C, Javadian-Elyaderani P, Schweyer S, Schutte D, Shoukier M, Karimi-Busheri F, Weinfeld M, Rasouli-Nia A, Hengstler JG, Mantilla A, Soleimanpour-Lichaei HR, Engel W (2010). Pathways of proliferation and antiapoptosis driven in breast cancer stem cells by stem cell protein piwil2. Cancer Res.

[11] Lee JH, Schutte D, Wulf G, Fuzesi L, Radzun HJ, Schweyer S, Engel W, Nayernia K (2006). Stem-cell protein Piwil2 is widely expressed in tumors and inhibits apoptosis through activation of Stat3/Bcl-XL pathway. Hum Mol Genet.

[12] Vourekas A, Zheng Q, Alexiou P, Maragkakis M, Kirino Y, Gregory BD, Mourelatos Z (2012). Mili and Miwi target RNA repertoire reveals piRNA biogenesis and function of Miwi in spermiogenesis. Nat Struct Mol Biol.

[13] Yin DT, Wang Q, Chen L, Liu MY, Han C, Yan Q, Shen R, He G, Duan W, Li JJ, Wani A, Gao JX (2011). Germline stem cell gene PIWIL2 mediates DNA repair through relaxation of chromatin. PLoS One.

[14] Unhavaithaya Y, Hao Y, Beyret E, Yin H, Kuramochi-Miyagawa S, Nakano T, Lin H (2009). MILI, a PIWI-interacting RNA-binding protein, is required for germ line stem cell self-renewal and appears to positively regulate translation. J Biol Chem.

[15] Aravin A, Gaidatzis D, Pfeffer S, Lagos-Quintana M, Landgraf P, Iovino N, Morris P, Brownstein MJ, Kuramochi-Miyagawa S, Nakano T, Chien M, Russo JJ, Ju J (2006). A novel class of small RNAs bind to MILI protein in mouse testes. Nature.

[16] Aravin AA, Sachidanandam R, Girard A, Fejes-Toth K, Hannon GJ (2007). Developmentally regulated piRNA clusters implicate MILI in transposon control. Science.

[17] Gao JX, Zhou Q (2009). Epigenetic progenitors in tumor initiation and development. Drug Discovery Today: Dis Models.

[18] Yin H, Lin H (2007). An epigenetic activation role of Piwi and a Piwi-associated piRNA in Drosophila melanogaster. Nature.

[19] Cox DN, Chao A, Baker J, Chang L, Qiao D, Lin H (1998). A novel class of evolutionarily conserved genes defined by piwi are essential for stem cell self-renewal. Genes Dev.

[20] Liu JJ, Shen R, Chen L, Ye Y, He G, Hua K, Jarjoura D, Nakano T, Ramesh GK, Shapiro CL, Barsky SH, Gao JX (2010). Piwil2 is expressed in various stages of breast cancers and has the potential to be used as a novel biomarker. Int J Clin Exp Pathol.

[21] He G, Chen L, Ye Y, Xiao Y, Hua K, Jarjoura D, Nakano T, Barsky SH, Shen R, Gao JX (2010). Piwil2 expressed in various stages of cervical neoplasia is a potential complementary marker for p16. Am J Transl Res.

[22] Wang Y, Liu Y, Shen X, Zhang X, Chen X, Yang C, Gao H (2012). The PIWI protein acts as a predictive marker for human gastric cancer. Int J Clin Exp Pathol.

[23] Yazarloo F, Shirkoohi R, Mobasheri MB, Emami A, Modarressi MH (2013). Expression analysis of four testis-specific genes AURKC, OIP5, PIWIL2 and TAF7L in acute myeloid leukemia: a gender-dependent expression pattern. Med Oncol.

[24] Oh SJ, Kim SM, Kim YO, Chang HK (2012). Clinicopathologic implications of PIWIL2 expression in colorectal cancer. Korean J Pathol.

[25] Li L, Yu C, Gao H, Li Y (2010). Argonaute proteins: potential biomarkers for human colon cancer. BMC Cancer.

[26] Wang QE, Han C, Milum K, Wani AA (2011). Stem cell protein Piwil2 modulates chromatin modifications upon cisplatin treatment. Mutat Res.

[27] Gainetdinov IV, Skvortsova YV, Stukacheva EA, Bychenko OS, Kondratieva SA, Zinovieva MV, Azhikina TL (2014). Expression profiles of PIWIL2 short isoforms differ in testicular germ cell tumors of various differentiation subtypes. PLoS One.

[28] Gao JX, Dakubo G (2011). Development of Tumor Stem Cells: Implication in Field Cancerization. Field Cancerization: Basic Science and Clinical Applications.

[29] Shi RR, Liu J, Zou Z, Qi YM, Zhai MX, Zhai WJ, Gao YF (2013). The immunogenicity of a novel cytotoxic T lymphocyte epitope from tumor antigen PL2L60 could be enhanced by 4-chlorophenylalanine substitution at position 1. Cancer Immunol Immunother.

[30] Lumbreras V, Campos N, Boronat A (1995). The use of an alternative promoter in the Arabidopsis thaliana HMG1 gene generates an mRNA that encodes a novel 3-hydroxy-3-methylglutaryl coenzyme A reductase isoform with an extended N-terminal region. Plant J.

[31] Ayoubi TA, Van De Ven WJ (1996). Regulation of gene expression by alternative promoters. FASEB J.

[32] Dieci G, Fiorino G, Castelnuovo M, Teichmann M, Pagano A (2007). The expanding RNA polymerase III transcriptome. Trends Genet.

[33] Consortium TF, Carninci P, Kasukawa T, Katayama S, Gough J, Frith MC, Maeda N, Oyama R, Ravasi T, Lenhard B, Wells C, Kodzius R, Shimokawa K (2005). The transcriptional landscape of the mammalian genome. Science.

[34] Carninci P, Sandelin A, Lenhard B, Katayama S, Shimokawa K, Ponjavic J, Semple CA, Taylor MS, Engstrom PG, Frith MC, Forrest AR, Alkema WB, Tan SL (2006). Genome-wide analysis of mammalian promoter architecture and evolution. Nat Genet.

[35] Kim TH, Barrera LO, Zheng M, Qu C, Singer MA, Richmond TA, Wu Y, Green RD, Ren B (2005). A high-resolution map of active promoters in the human genome. Nature.

[36] Kapranov P, Drenkow J, Cheng J, Long J, Helt G, Dike S, Gingeras TR (2005). Examples of the complex architecture of the human transcriptome revealed by RACE and high-density tiling arrays. Genome Res.

[37] Kimura K, Wakamatsu A, Suzuki Y, Ota T, Nishikawa T, Yamashita R, Yamamoto JI, Sekine M, Tsuritani K, Wakaguri H, Ishii S, Sugiyama T, Saito K (2006). Diversification of transcriptional modulation: large-scale identification and characterization of putative alternative promoters of human genes. Genome Res.

[38] Yoo EJ, Cooke NE, Liebhaber SA (2013). Identification of a secondary promoter within the human B cell receptor component gene hCD79b. J Biol Chem.

[39] Kowalczyk MS, Hughes JR, Garrick D, Lynch MD, Sharpe JA, Sloane-Stanley JA, McGowan SJ, De Gobbi M, Hosseini M, Vernimmen D, Brown JM, Gray NE, Collavin L (2012). Intragenic enhancers act as alternative promoters. Mol Cell.

[40] Heyn H, Ferreira HJ, Bassas L, Bonache S, Sayols S, Sandoval J, Esteller M, Larriba S (2012). Epigenetic disruption of the PIWI pathway in human spermatogenic disorders. PLoS One.

[41] Chen Y, Hu W, Lu Y, Jiang S, Li C, Chen J, Tao D, Liu Y, Yang Y, Ma Y (2014). A TALEN-based specific transcript knock-down of PIWIL2 suppresses cell growth in HepG2 tumor cell. Cell Prolif.

[42] Jiang S, Zhao L, Lu Y, Wang M, Chen Y, Tao D, Liu Y, Sun H, Zhang S, Ma Y (2014). Piwil2 inhibits keratin 8 degradation through promoting p38-induced phosphorylation to resist Fas-mediated apoptosis. Mol Cell Biol.

[43] Yao Y, Li C, Zhou X, Zhang Y, Lu Y, Chen J, Zheng X, Tao D, Liu Y, Ma Y (2014). PIWIL2 induces c-Myc expression by interacting with NME2 and regulates c-Myc-mediated tumor cell proliferation. Oncotarget.

[44] Lu Y, Zhang K, Li C, Yao Y, Tao D, Liu Y, Zhang S, Ma Y (2012). Piwil2 suppresses P53 by inducing phosphorylation of signal transducer and activator of transcription 3 in tumor cells. PLoS One.

[45] Zhang K, Lu Y, Yang P, Li C, Sun H, Tao D, Liu Y, Zhang S, Ma Y (2012). HILI inhibits TGF-beta signaling by interacting with Hsp90 and promoting TbetaR degradation. PLoS One.

[46] Li D, Sun X, Yan D, Huang J, Luo Q, Tang H, Peng Z (2012). Piwil2 modulates the proliferation and metastasis of colon cancer via regulation of matrix metallopeptidase 9 transcriptional activity. Exp Biol Med (Maywood).

[47] Ihle JN (1996). STATs: signal transducers and activators of transcription. Cell.

[48] Darnell JE (1997). STATs and gene regulation. Science.

[49] Bromberg JF, Wrzeszczynska MH, Devgan G, Zhao Y, Pestell RG, Albanese C, Darnell JE (1999). Stat3 as an oncogene. Cell.

[50] Bowman T, Garcia R, Turkson J, Jove R (2000). STATs in oncogenesis. Oncogene.

[51] Linher-Melville K, Singh G (2014). The transcriptional responsiveness of LKB1 to STAT-mediated signaling is differentially modulated by prolactin in human breast cancer cells. BMC Cancer.

[52] Huang M, Page C, Reynolds RK, Lin J (2000). Constitutive activation of stat 3 oncogene product in human ovarian carcinoma cells. Gynecol Oncol.

[53] Burke WM, Jin X, Lin HJ, Huang M, Liu R, Reynolds RK, Lin J (2001). Inhibition of constitutively active Stat3 suppresses growth of human ovarian and breast cancer cells. Oncogene.

[54] Catlett-Falcone R, Dalton WS, Jove R (1999). STAT proteins as novel targets for cancer therapy. Signal transducer an activator of transcription. Curr Opin Oncol.

[55] Turkson J (2004). STAT proteins as novel targets for cancer drug discovery. Expert Opin Ther Targets.

[56] Waters KM, Sontag RL, Weber TJ (2013). Hepatic leukemia factor promotes resistance to cell death: implications for therapeutics and chronotherapy. Toxicol Appl Pharmacolxs.

[57] Falvey E, Fleury-Olela F, Schibler U (1995). The rat hepatic leukemia factor (HLF) gene encodes two transcriptional activators with distinct circadian rhythms, tissue distributions and target pReferences. EMBO J.

[58] Gachon F, Olela FF, Schaad O, Descombes P, Schibler U (2006). The circadian PAR-domain basic leucine zipper transcription factors DBP, TEF, and HLF modulate basal and inducible xenobiotic detoxification. Cell Metab.

[59] Inaba T, Inukai T, Yoshihara T, Seyschab H, Ashmun RA, Canman CE, Laken SJ, Kastan MB, Look AT (1996). Reversal of apoptosis by the leukaemia-associated E2A-HLF chimaeric transcription factor. Nature.

[60] Inaba T, Roberts WM, Shapiro LH, Jolly KW, Raimondi SC, Smith SD, Look AT (1992). Fusion of the leucine zipper gene HLF to the E2A gene in human acute B-lineage leukemia. Science.

[61] Dieci G, Giuliodori S, Catellani M, Percudani R, Ottonello S (2002). Intragenic promoter adaptation and facilitated RNA polymerase III recycling in the transcription of SCR1, the 7SL RNA gene of Saccharomyces cerevisiae. J Biol Chem.

