# Supplementary material for: An unusual intragenic promoter of PIWIL2 contributes to aberrant activation of oncogenic PL2L60
Source: Oncotarget. 2017 May 2;8(28):46104–20. doi: 10.18632/oncotarget.17553 (PMC5542253; doi:10.18632/oncotarget.17553)
Supplement: Supplementary file 1 [file oncotarget-08-46104-s001.pdf]

An unusual intragenic promoter of *PIWIL2* contributes to aberrant activation of oncogenic *PL2L60*

SUPPLEMENTARY TABLES

Supplementary Table 1: Primers for screening and refining promoter.

See Supplementary File 1

Supplementary Table 2: Primers for site-directed point mutations

Primer pairs Fragment -2380/2330	Primer sequence	Restriction Enzyme site	Product size
OCT1-For	5'- GGGGTACCTTGAAGGATCCAAGAAATCA-3'	Kpn1	50bp
OCT1-Rev	5'-CCCAAGCTTGGATCGCAGATCTAAATATGAGTCAA -3'	Hind3	
BCL6-For	5'-GGGGTACCTTGTCATACATGAGAAATCA -3'	Kpn1	50bp
BCL6-Rev	5'-CCCAAGCTTGGATCGCAGATCTAAATATGAGTCAA -3'	Hind3	
STAT(-)-For	5'-GGGGTACCTTGTCATATCCACCAGATCA -3'	Kpn1	50bp
STAT(-)-Rev	5'-CCCAAGCTTGGATCGCAGATCTAAATATGAGTCAA -3'	Hind3	
STAT(+)-For	5'- GGGGTACCTTGTCATGCATAAGAAATCA -3'	Kpn1	50bp
STAT(+)-Rev	5'-CCCAAGCTTGGATCGCAGATCTAAATATGAGTCAA -3'	Hind3	
MEIS1(GFI1)-For	5'-GGGGTACCTTGTCATATCCAAGATCCGATTGCCTAA -3'	Kpn1	50bp
MEIS1(GFI1)-Rev	5'- CCCAAGCTTGGATCGCAGATCTAAATATGAGTCAA-3'	Hind3	
HLF(m)-For	5'-GGGGTACCTTGTCATATCCAAGAAATCATTGCAGCCCTCGAGGT-3'	Kpn1	50bp
HLF(m)-Rev	5'- CCCAAGCTTGGATCGCAGATCTAAATATGAGTCAA-3'	Hind3	

Note: Site-directed point mutations were performed on fragment -2380/-2330 using the primers, resulting 50 bp sequences with mutated binding sites (refer to Figure 3E and Supplementary Table 7).

Supplementary Table 3: Primers for ChIP-DNA PCR

Primer pairs	Primer sequence	Product size
ChIP-For	5'-CCCAGTGGTCTCTTTTAAGGAGTAG-3'	245bp
ChIP-Rev	5'-ACATGTAACAAAAGGAACTCAGACC-3'	

Note: DNA PCR was performed after ChIP with antibodies to STAT3, HLF or Pol II. The sequence of DNA-PCR product is as follow: CCCAGTGGTCTCTTTTAAGGAGTAGACATTTTTAATTCTGATGAAGTAGAATTTATACTTTTTCTTTCAT AGGTTTTGGTTTTGTTGTCATATCCAAGAAATCATTGCCTAACTCGAGGTCGCAAAGATTTTCTGTTTTTTTTTTTA GGTTTATAGTTTTGACTCATATTTAGATCTGCGATCCATTTTGAGTTAACTTTTACAGGTGATGTCAGGTAACGG TCTGAGTTCCTTTTGTTACATGT. Green font indicates the core of 50 nt with promoter activity. The fragment is located from 22361081 to 22361135 of *Homo sapiens* chromosome 8 in alternate assembly CHM1_1.1, or from 26592 to 26841 of *PIWIL2* gene.

Supplementary Table 4: Primers for real-time PCR

Primer pairs	Primer sequence	Product size
STAT3-For	5’-ATACTTCTGATTCTGGCTTCCTTCC-3’	171 bp
STAT3-Rev	5’-TGTCCAACCTGTAACTCTCTCCCC-3’	
HLF-For	5’-CCCTCGGTCATGGACCTCA-3’	126 bp
HLF-Rev	5’- ACTTGGTGTATTGCGGTTTGC-3’	

Supplementary Table 5: Primers for GEM RT-PCR

Primer pairs	Primer sequence	Product size
Exon 1-3-For	5’-CTCAGTGGCACCAGACCTAAAA-3’	387bp
Rev	5’-AAGGAGGCACTTCTCGTTTTGA-3’	
Exon 3-6-For	5’-TCCTTCAAAACGAGAAGTGCCT-3’	430bp
Rev	5’-GTTCAGTCCCAAAGACTGAGGA-3’	
Exon 6-14-For	5’-TCCTCAGTCTTTGGGACTGAACCT-3’	1015bp
Rev	5’-GACATCTTTATGCAGACTGAGCCC-3’	
Exon 13-21-For	5’-AGATGAAGAAGGACTTCAGGGCCA-3’	1041bp
Rev	5’-GAACTACAAACACCACCATCTTGGG-3’	
Exon 21-23-For	5’-TTGTGGTGTACCGAGATGGAGTGT-3’	873bp
Rev	5’-CAGCGGCTACCTACAAACTTGCTT-3’	
Exon 18-21-For	5’-TGAACTGTAAACTGGGTGGTGAGC-3’	496bp
Rev	5’-CACTCACAGCTGGTTATGGTATGA-3’	
β-actin-For	5’-TGAACCCTAAGGCCAACCGTGAAA-3’	136bp
Rev	5’-GAGTCCATCACAATGCCTGTGGTA-3’	

Supplementary Table 6: Sequences of siRNA

Targets	siRNA sequence	Target region
siE7For	5’-GCCTGTTAAGCTTCAACAATT-3’	Exon 7
Rev	5’-TTGTTGAAGCTTAACAGGCTT-3’	
siE21For	5’-CUAUGAGAUUCCUCAACUACAGAAG-3’	Exon 21
Rev	5’-CUUCUGUAGUUGAGGAAUCUCAUAGUU-3’	
siHLF For	5’-GCAAUACACCAAGUCCCAUTT -3’	HLF
Rev	5’-AUGGGACUUGGUGUAUUGCTT-3’	
siNC For	5’-UUCUCCGAACGUGUCACGUTT-3’	Scrable
Rev	5’-ACGUGACACGUUCGGAGAATT-3’	

Supplementary Table 7: Binding sequences before and after site-directed point mutation

OCT1	TTGTCATATCCAAGAAATCA
OCT1(mut)	TTGAAGGATCCAAGAAATCA
BCL6	TTGTCATATCCAAGAAATCA
BCL6(mut)	TTGTCATACATGAGAAATCA
STAT(-)	TTGTCATATCCAAGAAATCA
STAT(-)(mut)	TTGTCATATCCACCAGATCA
STAT(+)	TTGTCATATCCAAGAAATCA
STAT(+)(mut)	TTGTCATGCATAAGAAATCA
MEIS1(GFI1)	TTGTCATATCCAAGAAATCATTGCCTAA
MEIS1(GFI1)(mut)	TTGTCATATCCAAGATCCGATTGCCTAA
HLF	TTGTCATATCCAAGAAATCATTGCCTAACTCGAGGT
HLF(mut)	TTGTCATATCCAAGAAATCATTGCAGCCCTCGAGGT

Note: Red fonts indicate the binding sites or the nucleotides replaced. Mut: mutated.
