# Supplementary material for: An unusual intragenic promoter of PIWIL2 contributes to aberrant activation of oncogenic PL2L60
Source: Oncotarget. 2017 May 2;8(28):46104–20. doi: 10.18632/oncotarget.17553 (PMC5542253; doi:10.18632/oncotarget.17553)
Supplement: Supplementary file 2 [file oncotarget-08-46104-s002.docx]

**Supplementary** **Table 1: Primers for screening and refining promoter**

| **Primer pairs**  **Initial segments** | **Primer sequence** | **Restriction**  **Enzyme site** | **Product**  **size** |
| --- | --- | --- | --- |
| -2409/+155-For | 5'- GGTACCGGCTGTTCTTGAACTCCTGG-3' | Kpn1 | 2564bp |
| -2409/+155-Rev | 5'- GCTAGCATGTGATCTCTTTCCCATCAGACA-3' | Nhe1 |  |
| -3688/-2280-For | 5'-GGTACCGTAGTGTTCCATTTTGTGGCTCTAC-3' | Kpn1 | 1408bp |
| -3688/-2280-Rev | 5'-AAGCTTGGATCGCAGATCTAAATATGAGTCAA-3' | Hind3 |  |
| -6228/-3633-For | 5'-GGTACCAAAGGCCTAAATTGGTATGAAGCCT-3' | Kpn1 | 2595bp |
| -6228/-3633-Rev | 5'-AAGCTTGTAGAGCCACAAAATGGAACACTAC-3' | Hind3 |  |
| -8673/-6193-For | 5'-GGTACCTTAAGCTCAAATTCACACCGAGTTG-3' | Kpn1 | 2480bp |
| -8673/-6193-Rev | 5'-AAGCTTAAGCCAGATAAGGCTTCATACCAAT-3' | Hind3 |  |
| -11225/-8643-For | 5'-GGTACCTTTGGCTATTATGTGTCTCTCCTGA-3' | Kpn1 | 2582bp |
| -11225/-8643-Rev | 5'-AAGCTTTTACACAACTCGGTGTGAATTTGAG-3' | Hind3 |  |
| -13707/-11190-For | 5'-GGTACCGAGTGAATGGTGGGGTCTATCTTTA-3' | Kpn1 | 2517bp |
| -13707/-11190-Rev  **Enhancer assay**  **-3688/-2280** | 5'-CCCGGGTTCTGAGAAATCAGGAGAGACACAT-3' | Sma1 |  |
| U-F/R-For | 5'-GGTACCGTAGTGTTCCATTTTGTGGCTCTAC-3' | Kpn1 | 1408bp |
| U-F/R-Rev | 5'-GGTACCGGATCGCAGATCTAAATATGAGTCAA-3' | Kpn1 |  |
| D-F/R-For | 5'-TCTAGAGTAGTGTTCCATTTTGTGGCTCTAC-3' | Xba1 | 1408bp |
| D-F/R-Rev  **Subfragments of -3688/-2280** | 5'-TCTAGAGGATCGCAGATCTAAATATGAGTCAA-3' | Xba1 |  |
| PGL3-5.1-For | 5'-GGTACCGTAGTGTTCCATTTTGTGGCTCTAC-3' | Kpn1 | 729bp |
| PGL3-5.1-Rev | 5'-AAGCTTGAGGTGAGAGGACTGCTTGA-3' | Hind3 |  |
| PGL3-5.2.1-For | 5'-GGTACCACATCTCACTGCAGCCTTGG-3' | Kpn1 | 329bp |
| PGL3-5.2.1-Rev  PGL3-5.2.2 -For  PGL3-5.2.2-Rev  **50-nt Fragments of -2609/-2280**  PGL3-50bp-For  PGL3-50bp -Rev  PGL3-100bp-For  PGL3-100bp-Rev  PGL3-150bp-For  PGL3-150bp -Rev  PGL3-200bp-For  PGL3-200bp -Rev  PGL3-250bp-For  PGL3-250bp-Rev | 5'-AAGCTTTGGCGCATGCCTGTAGTCCC-3'  5'- GGTACCCCACACACCTGGCTGTTTTG-3'  5'- AAGCTTGGATCGCAGATCTAAATATGAGTCAA-3'  5'-GGGGTACCGTTTTTTTTTTTAGGTTTATAG -3'  5'- CCCAAGCTTGGATCGCAGATCTAAATATGAGTCAA-3'  5'-GGGGTACCTTGTCATATCCAAGAAATCA -3'  5'-CCCAAGCTTGGATCGCAGATCTAAATATGAGTCAA-3'  5'- GGGGTACCATTCTGATGAAGTAGAATTTA-3'  5'-CCCAAGCTTGGATCGCAGATCTAAATATGAGTCAA-3'  5'-GGGGTACCGAGCCACCATGCCCGGCCCA-3'  5'-CCCAAGCTTGGATCGCAGATCTAAATATGAGTCAA -3'  5'- GGGGTACCTCAAGTGATCCCCCCGCCTCA-3'  5'-CCCAAGCTTGGATCGCAGATCTAAATATGAGTCAA-3' | Hind3  Kpn1  Hind3  Kpn1  Hind3  Kpn1  Hind3  Kpn1  Hind3  Kpn1  Hind3  Kpn1  Hind3 | 400bp  50bp  100bp  150bp  200bp  250bp |

Note: All the primer templates in all supplementary tables are from chromosome 8 of *Homo Sapiens* in alternate assembly CHM1_1.1 (**NC_018919.2)**. Initial segments: used for screening putative promoter activity; enhancer assay: used to exclude enhancer activity of a putative promoter (**-3688/-2280)**; Sub-fragments of **-3688/-2280**: used to refine promoter activity; 50-nt fragments: used to define regulatory cis-elements. Restriction enzyme sites: enzyme sensitive sites on the pGBL3-basic leuciferase report vector (also seen in other tables). Product size: size of nt fragments inserted into pGL3Basic vectors.
